# GPS-CCD: A Novel Computational Program for the Prediction of Calpain Cleavage Sites

**DOI:** 10.1371/journal.pone.0019001

**Published:** 2011-04-20

**Authors:** Zexian Liu, Jun Cao, Xinjiao Gao, Qian Ma, Jian Ren, Yu Xue

**Affiliations:** 1 Hefei National Laboratory for Physical Sciences at Microscale and School of Life Sciences, University of Science and Technology of China, Hefei, China; 2 Hubei Bioinformatics and Molecular Imaging Key Laboratory, Department of Systems Biology, College of Life Science and Technology, Huazhong University of Science and Technology, Wuhan, Hubei, China; 3 State Key Laboratory of Biocontrol, School of Life Sciences, Sun Yat-sen University (SYSU), Guangzhou, Guangdong, China; Kyushu Institute of Technology, Japan

## Abstract

As one of the most essential post-translational modifications (PTMs) of proteins, proteolysis, especially calpain-mediated cleavage, plays an important role in many biological processes, including cell death/apoptosis, cytoskeletal remodeling, and the cell cycle. Experimental identification of calpain targets with *bona fide* cleavage sites is fundamental for dissecting the molecular mechanisms and biological roles of calpain cleavage. In contrast to time-consuming and labor-intensive experimental approaches, computational prediction of calpain cleavage sites might more cheaply and readily provide useful information for further experimental investigation. In this work, we constructed a novel software package of GPS-CCD (Calpain Cleavage Detector) for the prediction of calpain cleavage sites, with an accuracy of 89.98%, sensitivity of 60.87% and specificity of 90.07%. With this software, we annotated potential calpain cleavage sites for hundreds of calpain substrates, for which the exact cleavage sites had not been previously determined. In this regard, GPS-CCD 1.0 is considered to be a useful tool for experimentalists. The online service and local packages of GPS-CCD 1.0 were implemented in JAVA and are freely available at: http://ccd.biocuckoo.org/.

## Introduction

Calpains constitute an important family of the Ca^2+^-dependent cysteine proteases, which contain a nucleophilic cysteine in the catalytically active site [1]–[7]. Calpains are widely expressed in mammalians and conserved across eukaryotes [1]–[5], [8], [9]. For instance, in budding yeast, at least one calpain-like protease, Rim13/Cpl1, has been identified, although its functions are still elusive [8], [9]. In humans, there are over 14 distinct members of the calpain superfamily, some of which are tissue specific. Calpain 1 (µ-calpain, micromolar Ca^2+^-requiring) and Calpain 2 (m-calpain, millimolar Ca^2+^-requiring) are ubiquitously expressed and well characterized isoforms [1], [2], [4], [5]. Through spatial and temporal cleavage of a variety of substrates to change their conformation, function and stability [1]–[4], Ca^2+^-activated calpains play an important role in numerous biological processes, including the regulation of gene expression, signal transduction, cell death/apoptosis, remodeling cytoskeletal attachments during cell fusion/motility and cell cycle progression [1]–[4], [6], [10]–[12]. Moreover, calpain aberrancies are frequently implicated in a variety of diseases and cancers [5]–[7], [13], [14]. Although many studies have tried to dissect the regulatory roles and molecular mechanisms of calpain-dependent cleavage, in fact our understanding of calpain is still fragmentary.

Identification of the site-specific calpain substrates is fundamental for dissecting the roles of calpain cleavage in numerous biological pathways. Besides the conventional experimental approaches with Edman N-terminal sequencing or mass spectrometry (MS) [12], [15], a peptide library approach was also designed to investigate the sequence/structural specificities of calpains [16]–[18]. Thus far, hundreds of calpain-cleaved proteins have been experimentally identified, including structural proteins, membrane receptors, and transcription factors [12], [15]–[18]. However, high-throughout technique for the identification of calpain substrates is still limited. Recently, besides time-consuming and labor-intensive experimental methods, the development of computational approaches has also promoted the discovery of the proteolytic cleavage sites [16], [19]–[22]. In a previous study [16], Tompa *et al*. collected 106 calpain cleavage sites in 49 substrates from the scientific literature, and determined the amino acid preferences around the cleavage bond, from P_4_ (upstream) to P_7_' (downstream). They constructed a position-specific scoring matrix (PSSM), and observed that the preferred residues for µ- calpain and m- calpain recognition are Leu, Thr and Val in the P_2_ position, and Lys, Tyr and Arg in the P_1_ position [16]. Based on this rationale, they synthesized a short peptide of TPLK|SPPPSPR (“|” is the potentially cleaved position), which was experimentally verified to be a superior substrate of calpain [16]. With a similar algorithm, Boyd *et al*. developed an online tool of PoPS (Prediction of Protease Specificity), which allows researchers to use their own training data for building computational models and predicting protease specificity [19], [20]. Based on the frequency and substitution matrix scoring strategy, SitePrediction was designed for predicting Calpain 1 and 2 specific cleavage sites, respectively [21]. Recently, duVerle *et al*. also constructed a web service for the prediction of calpain cleavage sites [22]. Although a number of predictors were implemented, more efforts need to be made for further improving the prediction accuracy.

In this work, we collected 368 experimentally verified calpain cleavage sites in 130 proteins (Supplementary Table S1). With a previously released algorithm of GPS (Group-based Prediction System) [23], we developed a novel software package of GPS-CCD (Calpain Cleavage Detector) for the prediction of calpain cleavage sites. The leave-one-out validation and 4-, 6-, 8-, 10-fold cross-validations were performed to evaluate the performance of the prediction system. By comparison, the GPS 2.0 algorithm was employed for its outstanding prediction performance, with an accuracy 89.98%, sensitivity 60.87% and specificity 90.07%. Furthermore, there are many proteins experimentally identified as calpain substrates for which the exact cleavage sites have not been verified, and we collected 196 such proteins from PubMed (Supplementary Table S2). As an application, we predicted potential calpain cleavage sites for these targets (Supplementary Table S2). These prediction results might be a useful resource for further experimental investigation. Finally, the online service and local packages of GPS-CCD 1.0 were implemented in JAVA 1.5 (J2SE 5.0) and are freely available for academic researchers at: http://ccd.biocuckoo.org/.

## Methods

### Data preparation

We searched the scientific literature from PubMed with the keyword of “calpain” to obtain the experimentally verified calpain substrates with cleavage sites (before June 30^th^, 2010). The data collected by Tompa *et al.* and duVerle *et al.* were also integrated [16], [22], while the protein sequences were retrieved from the UniProt database.

We defined a *calpain cleavage peptide* CCP(*m*, *n*) as a cleavage bond flanked by *m* residues upstream and *n* residues downstream. As previously described [23], [24], we regarded all experimentally verified cleavage sites as positive data (+), while all other non-cleavage sites in the same substrates were taken as negative data (−). If a cleavage site locates at the N- or C-terminus of the protein and the length of the peptide is smaller than *m*+*n*, we added one or multiple “*” characters as pseudo amino acids to complement the CCP(*m*, *n*). The positive data (+) set for training might contain several homologous sites from homologous proteins. If the training data were highly redundant with too many homologous sites, the prediction accuracy would be overestimated. To avoid such overestimation, we clustered the protein sequences with a threshold of 40% identity by CD-HIT [25]. If two proteins were similar with ≥40% identity, we re-aligned the proteins with BL2SEQ, a program in the BLAST package [26], and checked the results manually. If two calpain cleavage sites from two homologous proteins were at the same position after sequence alignment, only one item was preserved, the other was discarded. Finally, the non-redundant benchmark data set for training contained 368 positive sites from 130 unique substrates (Supplementary Table S1).

### The algorithms

To predict the calpain cleavage sites, a previously self-developed GPS 2.0 algorithm was employed and improved [23]. Based on the hypothesis of similar short peptides exhibiting similar biological functions, we can use an amino acid substitution matrix, eg., BLOSUM62, to evaluate the similarity between two CCP(*m*, *n*). As previously described [23], the substitution score between two amino acids *a* and *b* can be denoted as Score (*a*, *b*). Then the similarity between two CCP(*m*, *n*) of *A* and *B* is defined as:
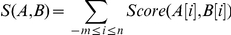



If S (*A, B*) <0, we simply redefined it as S (*A, B*)  = 0. A putative CCP(*m*, *n*) is compared with each of the experimentally verified cleavage peptides in a pairwise manner to calculate the similarity score. The average value of the substitution scores is regarded as the final score. Then we designed a motif length selection (MLS) approach to exhaustively test the combinations of CCP(*m*, *n*) (*m* = 1, …, 30; *n* = 1, …, 30). The optimal CCP(*m*, *n*) was selected for its highest leave-one-out performance. The *Sp* value was fixed at 90%.

Previously, we observed that different amino acid substitution matrices generated difference in the prediction [23]. To improve the robustness and performance of the prediction system, we developed the novel approach of “Matrix Mutation” (MaM) to generate an optimal or near-optimal matrix [23]. This method was also used in this work. First, BLOSUM62 was chosen as the initial matrix, while the leave-one-out validation was calculated. In BLOSUM62, the substitution score between “*” and other residues is −4 but redefined as 0. Then we fixed the specificity (*Sp*) at 90% to improve sensitivity (*Sn*) by randomly picking out one value from the BLOSUM62 matrix for mutation (+1 or −1). If the *Sn* value increased, the mutation was adopted. This process was terminated when the *Sn* value was not increased any further. The training order of MLS followed by MaM can not be reversed.

### Performance evaluation

As previously described [23], [24], four standard measurements, including accuracy (*Ac*), sensitivity (*Sn*), specificity (*Sp*) and Mathew correlation coefficient (*MCC*) were defined as shown below:
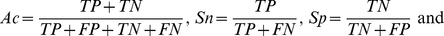


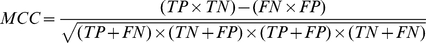



The self-consistency validation was calculated to evaluate the prediction performance on the benchmark data set. To further estimate the robustness of the prediction system, the leave-one-out validation and 4-, 6-, 8-, 10-fold cross-validations were also carried out. Receiver Operating Characteristic (ROC) curves and AROCs (area under ROCs) were performed.

### Implementation of the online service and local packages

The online service and local packages of GPS-CCD 1.0 were implemented in JAVA and are freely available at http://ccd.biocuckoo.org/. For the online service, we tested the GPS-CCD 1.0 on a variety of internet browsers, including Internet Explorer 6.0, Netscape Browser 8.1.3 and Firefox 2 under the Windows XP Operating System (OS), Mozilla Firefox 1.5 of Fedora Core 6 OS (Linux), and Safari 3.0 of Apple Mac OS X 10.4 (Tiger) and 10.5 (Leopard). For the Windows and Linux systems, the latest version of the Java Runtime Environment (JRE) package (JAVA 1.5 or later versions) of Sun Microsystems should be pre-installed. However, for Mac OS, GPS-CCD 1.0 can be directly used without any additional packages. For convenience, we also developed local packages of GPS-CCD 1.0, which worked with the three major Operating Systems, Windows, Linux and Mac.

## Results

### Development of GPS-CCD with the GPS 2.0 algorithm

**Figure 1:**
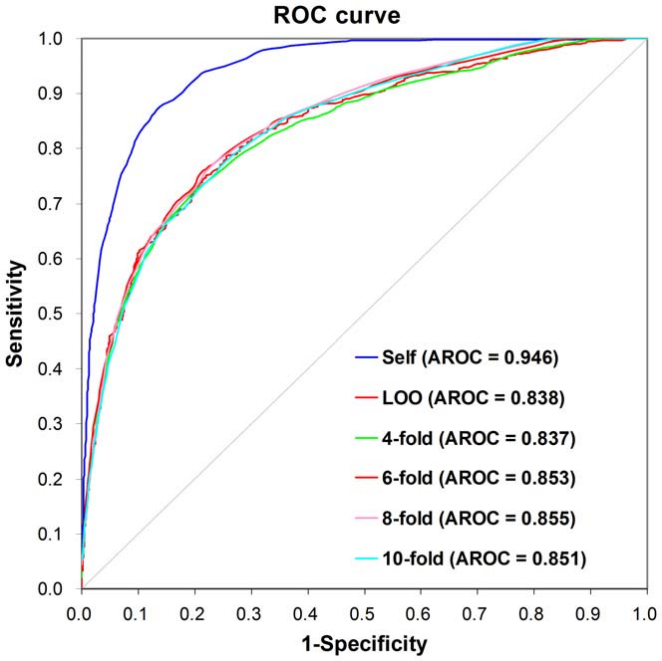
The prediction performance of GPS-CCD 1.0. The self-consistency validation, leave-one-out validation and 4-, 6-, 8-, 10-fold cross-validations were calculated. The Receiver Operating Characteristic (ROC) curves and AROC values were also performed.

In this work, we collected experimentally identified calpain cleavage sites from the scientific literature (Supplementary Table S1). By means of integration with previous studies and a simplification of redundancies, a dataset of 368 experimentally verified calpain cleavage sites in 130 proteins was constructed. Previously, we developed the GPS (Group-based Prediction System) algorithm for the prediction of phosphorylation sites [23], [24]. In contrast to the arbitrarily determined flanking peptides in our previous work [23], [24], here we exhaustively tested the combinations of CCP(*m*, *n*). The optimal CCP(10, 4) was selected for its highest leave-one-out performance. Then the scoring matrix BLOSUM62 was also optimized by MaM. After the training to improve performance, the self-consistency validation, the leave-one-out validation and 4-, 6-, 8-, 10-fold cross-validations were thoroughly carried out. ROC curves were drawn, and the AROC values were calculated as 0.946 (self-consistency), 0.838 (leave-one-out), 0.837 (4-fold), 0.853 (6-fold), 0.855 (8-fold) and 0.851 (10-fold), respectively (Figure 1). The self-consistency validation evaluates the prediction accuracy merely on the benchmark data, while the leave-one-out validation and 4-, 6-, 8-, 10-fold cross-validations assess the performance and robustness on an independent data set. Since the results of 4-, 6-, 8-, 10-fold cross-validations were close to the leave-one-out validation, we used the leave-one-out validation as the major performance indicator for further analysis.

**Figure 2:**
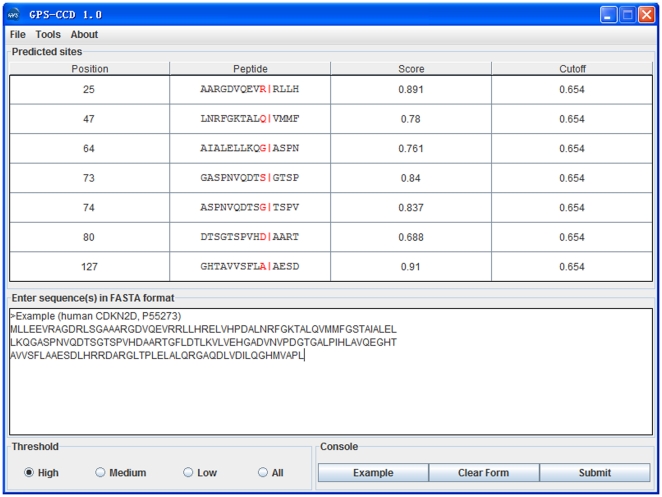
The screen snapshot of GPS-CCD software. A high threshold was chosen as the default cut-off. The human cyclin-dependent kinase 4 inhibitor D/CDKN2D (P55273) is presented as an example.

With this performance taken into consideration, we developed a novel predictor of GPS-CCD (Calpain Cleavage Detector). The *Ac*, *Sn* and *Sp* values of GPS-CCD with different cutoff values were presented (Table 1). To avoid too many false positive hits, a high threshold was chosen as the default threshold. As an example, the protein sequence of the human G1 cyclin-dependent kinase 4 inhibitor p19/CDKN2D/INK4d (UniProt ID: P55273) is presented (Figure 2). It was proposed that µ-calpain cleaves CDKN2D after the R25, H29, Q47, G64, L113 and A127 residues, and plays an important role in modulating cell cycle regulatory protein turnover [27]. With the default parameter (high threshold), we successfully predicted the four known bonds after R25, Q47, G64 and A127, with three additionally potential cleavage bonds after the S73, G74, and D80 residues (Figure 2).

**Table 1:** Comparison of the GPS 2.0 algorithm with other approaches.

Method	Threshold	*Ac*	*Sn*	*Sp*	*MCC*
GPS 2.0	High	94.87%	45.92%	95.01%	0.0998
	Medium	89.98%	60.87%	90.07%	0.0908
	Low	84.99%	66.58%	85.04%	0.0773
GPS 1.1		94.84%	34.51%	95.02%	0.0723
		89.74%	50.00%	89.86%	0.0706
		84.57%	60.33%	84.64%	0.0667
PoPS		94.70%	36.14%	94.90%	0.0817
		89.73%	52.45%	89.73%	0.0813
		84.73%	60.32%	84.82%	0.0731
SitePrediction 1^a^		94.77%	31.52%	94.95%	0.0645
		89.92%	41.30%	90.06%	0.0561
		84.97%	50.82%	85.07%	0.0539
SitePrediction 2^b^		94.72%	28.26%	94.92%	0.0563
		89.90%	39.67%	90.05%	0.0531
		84.87%	48.37%	84.97%	0.0500

For the construction of the GPS-CCD 1.0 software, the three thresholds of high, medium and low were chosen. We fixed the *Sp* values of GPS 2.0 to be identical or similar to other methods and compared the *Sn* values. The leave-one-out results were calculated for GPS 2.0, GPS 1.1 [24] and PoPS [19], [20]. The performance of SitesPrediction [21] was directly calculated.

*a*.Specific prediction of Calpain 1 cleavage sites;

*b*.Specific prediction of Calpain 2 cleavage sites.

### Comparison of different computational approaches

For comparison, we also investigated the performances of several other approaches or predictors, including GPS 1.1 algorithm [24], PoPS [19], [20], SitesPrediction [21] and CaMPDB [22]. The only difference between GPS 2.0 and GPS 1.1 is that the MaM process is not carried out in GPS 1.1. To avoid any bias, the same training data (368 sites) was used for GPS 1.1, while the CCP(10, 4) was determined with the highest leave-one-out result. Since the PoPS software package allows user-defined computational models [19], [20], we used our training data set to construct a PSSM model in PoPS. Again, the CCP(8, 3) was selected based on the highest leave-one-out result. The leave-one-out results of GPS 1.1 and PoPS were performed for comparison. Besides a frequency scoring algorithm, SitePrediction also adopted an additional substitution matrix scoring strategy by comparing potential cleavage sites to the known sites, and this method is quite similar with GPS 1.1 algorithm [21]. Since user-defined models can not be constructed in SitePrediction, we directly submitted the benchmark data set to calculate the performances of Calpain 1 (SitePrediction 1) and Calpain 2 (SitePrediction 2), respectively.

**Figure 3:**
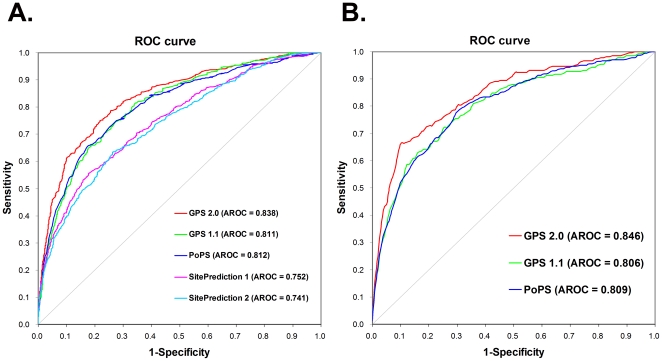
Comparison of GPS 2.0, GPS 1.1 [**24**], PoPS [**19]**, [20****], SitesPrediction [**21**] and CaMPDB [**22**]. The leave-one-out performances were calculated for GPS 2.0, GPS 1.1 and PoPS. We calculated the accuracy of SitesPrediction by directly submitting the benchmark data set for the prediction. (A) The data set contains 368 cleavage sites in 130 unique substrates; (B) For CaMPDB, we took 267 cleavage sites in 104 proteins from its website [22]. The highest AROC value in CaMPDB was 0.801.

In Table 1, we fixed the *Sp* values of GPS 1.1, PoPS and SitePrediction to be similar with GPS 2.0 and compared the *Sn* values. When the *Sp* value was ∼85%, the *Sn* values of GPS 2.0, GPS 1.1, PoPS, SitePrediction 1 and SitePrediction 2 were 66.58%, 60.33%, 60.32%, 50.82% and 48.37%, respectively (Table 1). Moreover, when the *Sp* value was ∼90%, the *Sn* values of GPS 2.0, GPS 1.1, PoPS, SitePrediction 1 and SitePrediction 2 were 60.87%, 50.00%, 52.45%, 41.30% and 39.67%, respectively (Table 1). In addition, when the *Sp* value was ∼95%, the *Sn* of GPS 2.0 (45.92%) was still much better than GPS 1.1 (34.51%), PoPS (36.14%), SitePrediction 1 (31.52%) and SitePrediction 2 (28.26%) (Table 1). Previously, it was observed that the accuracy of SitePrediction can be comparative with PoPS, when the same training and testing data sets were provided [21]. In our analysis, we confirmed this conclusion that the performance of SitePrediction like algorithm of GPS 1.1 is quite similar with PoPS (Table 1). The SitePrediction did not exhibit superior performance because of limited training data. Taken together, the prediction performance of the GPS 2.0 algorithm was much better than other methods. In addition, ROC curves were drawn, whereas the AROC value of the GPS 2.0 algorithm was generally better than the other approaches (Figure 3A).

In CaMPDB, duVerle *et al*. developed a calpain cleavage sites predictor with a training data set containing 267 cleavage sites in 104 proteins (http://www.calpain.org/prediction_view.rb) [22]. The tool always predicts 10 potential cleavage sites for any given protein sequences. If we divide one sequence into two fragments as inputs, the prediction results are different from the original sequence. Also, if we input a putative sequence as ‘AAAAAAAAAAA’, this program still provides 10 positive hits. In this regard, the *Ac*, *Sn*, *Sp* and *MCC* values can not be estimated. However, they calculated the AROCs of different methods, while the highest AROC was 0.801 for the Support Vector Machines (SVM) algorithms with Radial Basis Function (RBF) kernel [22]. To avoid any bias, we used the same data set (267 sites) for comparison. After training, the optimal CCP (8, 12) was determined for GPS 2.0 and GPS 1.1, while the CCP(6, 3) was selected for PoPS. Again, leave-one-out ROC curves were drawn, while AROC results were 0.846, 0.806, and 0.809 for GPS 2.0, GPS 1.1 and PoPS, respectively (Figure 3B). In this regard, the performances of GPS 1.1 and PoPS are similar with the previous study, while GPS 2.0 is much better.

### Large-scale prediction of calpain cleavage sites in proteins

**Figure 4:**
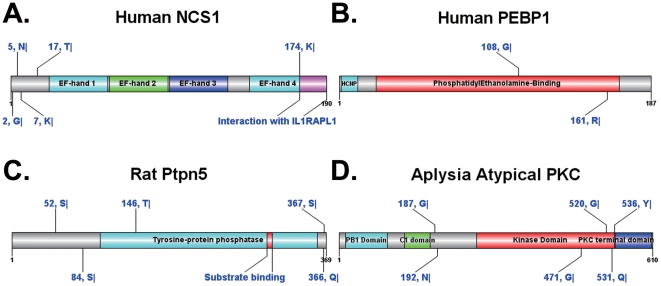
Applications of GPS-CCD 1.0. Here we predicted the potential calpain cleavage sites in the experimentally identified calpain substrates with a default threshold. (A) The human NCS1 (P62166); (B) The human PEBP (P30086); (C) The Rat Ptpn5 (P35234); (D) The *Aplysia* atypical PKC (C3VIX7).

While a large number of proteins have been experimentally verified to be cleaved by calpains, the *bona fide* cleavage sites still need to be elucidated. To perform an application of GPS-CCD 1.0, we first collected 196 calpain cleavage substrates from the scientific literature (Supplementary Table S2). With the default threshold (high), we predicted potentially calpain cleavage site for these proteins (Supplementary Table S2). The prediction results should be useful for further experimental verification. Several examples were randomly picked out, and their prediction results are presented in Figure 4 with the help of DOG 1.0 [28].

It was proposed that chronic exposure to paclitaxel (Taxol) activates µ-calpain and diminishes inositol trisphosphate (Ins*P*_3_)-mediated Ca^2+^ signaling, through cleaving and degrading neuronal calcium sensor-1/NCS1 (P62166) [29]. However, the precise cleavage sites have not been experimentally identified. Here, we predicted that the human NCS1 protein might be cleaved after G2, N5, K7, T17, and K174 (Figure 4A). Interestingly, most of these potential sites were located in the N-terminus of the protein, with the K174 site is at the boundary between the EF-hand 4 domain and the IL1RAPL1 Interaction domain. None of which are located within the EF-hand domain. As a serine protease inhibitor, human phosphatidylethanolamine-binding protein 1/PEBP (P30086) was identified as an *in vitro* and *in situ* calpain substrate, with the *bona fide* cleavage sites again not yet determined [30]. In a model of brain injury, activated calpain leads to PEBP degradation and enhances the chymostrypsin-like activity of the proteasome [30]. We predicted that PEBP might be cleaved after G108 and R161 (Figure 4B). Since both of the two sites locate in the phosphatidylethanolamine-binding domain, PEBP proteolysis by calpain might disrupt its original roles to alleviate impaired proteasome function in Alzheimer's disease (AD) [30]. Recent work by Xu *et al*. suggested that extrasynaptic NMDA receptors have an important role in excitotoxicity via the calpain-mediated cleavage of striatum-enriched protein-tyrosine phosphatase STEP/Ptpn5 (P35234) [31]. We predicted that STEP might be cleaved after S52, S84, T146, Q366 and S367 residues (Figure 4C). In addition, an atypical protein kinase C (C3VIX7) isolated from Aplysia californica was demonstrated to be a calpain substrate [32]. Here we predicted the cleavage bonds to be after G187, N192, G471, G520, Q531 and Y536 (Figure 4D).

## Discussion

Calpain-mediated cleavage is an important PTM of proteins [1]–[9]. The identification of new calpain substrates with cleavage sites is the key step to establishing a foundation for understanding the regulatory roles of the calpain cleavage processes. Although many studies have investigated the functions and biological roles of calpain cleavage in various cellular processes, an unambiguous consensus motif has still not been detected for either µ-calpain or m-calpain [16]–[18]. In contrast to labor-intensive and expensive experimental approaches, the computational prediction of calpain cleavage sites is comparatively simple, and might therefore be of great help in providing information for further experimental verification.

To date, hundreds of calpain cleavage sites were experimentally identified, while a large number of these known sites were collected in a variety of public databases [22], [33]–[35]. For example, a proteolytic event database of CutDB contains 63 known calpain substrates with 165 cleavage sites [33], whereas the peptidase database MEROPS has collected 101 Calpain 1 and 147 Calpain 2 sites, respectively [34], [35]. Also, a recently constructed database CaMPDB collected 104 experimental identified calpain targets with 267 sites by literature curation [22]. Based on these experimental data, several computational tools have developed for the prediction of calpain cleavages sites. For example, SitePrediction can distinguish between calpains, with a training data set containing 79 Calpain 1 and 103 Calpain 2 sites from MEROPS database [21]. Moreover, with 47 Calpain 1 and 57 Calpain 2 sites in *Homo sapiens*, SitePrediction also provides the organism-specific predictions [21]. In addition, several extra features for calpain cleavage sites prediction, such as PEST sequence (short peptide rich in Pro/P, Glu/E, Ser/S and Thr/T), solvent accessibility and secondary structure were considered and analyzed in PoPS and SitePrediction [19]–[21].

In this study, we presented a novel predictor of GPS-CCD with an improved GPS 2.0 algorithm [23]. In our benchmark data set, the number of experimentally identified calpain cleavage sites is still limited, while the specific calpain information for a considerable proportion of known sites is ambiguous. In this regard, GSP-CCD predictions didn't distinguish among different calpain isoforms as previously carried out [22]. By comparison, our approach is much better than other existing methods currently in use. Through the application of annotation, the exact cleavage sites for potential substrates identified in previous studies were obtained (Supplementary Table S2). In this regard, we conclude that GPS-CCD 1.0 is a useful tool for pinpointing potential calpain cleavage sites, while computational predictions followed by experimental verification should lead to an improved identification of calpain substrates in the near future.

With the continuous efforts that have led to the spate of reports, many functions have been assigned to calpains, with the result that the calpains target a broad range of broad substrates in a variety of biological processes. The collection of calpain substrates from the literature provided the opportunity to analyze the functional abundance and diversity of calpain cleavage processes. With a hypergeometric distribution [36], we statistically analyzed the enriched biological processes, molecular functions and cellular components with gene ontology (GO) annotations for the human calpain substrates (Supplementary Table S3). The GO association files were downloaded from the GOA database (EBI, on June 29^th^, 2010) [37]. For biological processes, our analysis suggests that calpain substrates are enriched in response to a variety of stimulus, such as drug (GO:0042493), corticosterone stimulus (GO:0051412), organic nitrogen (GO:0010243) and so on (Supplementary Table S3). Calpain cleavage is also highly implicated in regulation of mitochondrial membrane (GO:0046902, GO:0051881) and apoptosis (GO:0043066, GO:0042981, GO:0006916) (Supplementary Table S3). Also, the significantly over-represented molecular functions of human calpain substrates are protein activity and various molecular binding, which can be dynamically regulated by cleavage (Supplementary Table S3). Moreover, calpain cleavage targets were distributed in a variety of subcellular localizations, such as cytoplasm (GO:0005737), cytosol (GO:0005829), axon (GO:0030424), actin cytoskeleton (GO:0015629), and nucleoplasm (GO:0005654) (Supplementary Table S3). Taken together, our analysis can be a good start for further investigating molecular mechanisms of calpain cleavage.

## Supporting Information

Table S1 We collected 368 experimentally identified calpain cleavage sites in 130 unique proteins from the scientific literatures (PubMed). *a*. UniProt, the UniProt accession number; *b*. Position, the position of a calpain cleavage site, while its following bond can be disrupted by calpain; *c*. PMID, the primary references.
(XLS)

Table S2 From previous experimental studies, we also collected 196 calpain cleavage substrates. The exact calpain cleavage sites had not yet been experimentally determined. The default threshold (high) was adopted for GPS-CCD 1.0.
(XLS)

Table S3 The top 15 most enriched processes, functions and localizations of human calpain cleavage substrates. From Table S1 and Table S2, we collected 176 human calpain targets. The human proteome contains 18,262 proteins which have at least one GO term. *a*. the number of proteins annotated; *b*. the proportion of proteins annotated; *c*. E-ratio, enrichment ratio.
(XLS)
